# Pharmacy Practice and Education in Latvia

**DOI:** 10.3390/pharmacy6010009

**Published:** 2018-01-20

**Authors:** Ruta Muceniece, Una Riekstina, Baiba Maurina, Vija Enina, Jeffrey Atkinson

**Affiliations:** 1 Faculty of Medicine, the University of Latvia, 19 Raina Blvd., LV-1001 Riga, Latvia; Ruta.Muceniece@lu.lv (R.M.); Una.Riekstina@lu.lv (U.R.); 2 Faculty of Pharmacy, Riga Stradins University, 16 Dzirciema Street, LV-1007 Riga, Latvia; Baiba.Maurina@rsu.lv (B.M.); vija.enina@inbox.lv (V.E.); 3 Pharmacolor Consultants Nancy, 12 rue de Versigny, 54600 Villers, France

**Keywords:** pharmacy, education, practice, Latvia, European Union

## Abstract

The PHARMINE (“*Pharmacy Education in Europe*”) project studied the organisation of pharmacy practice and education in the member states of the European Union (EU). The work was carried out using an electronic survey sent to chosen pharmacy representatives. The surveys of the individual member states are now being published as reference documents. This paper presents the results of the PHARMINE survey on pharmacy practice and education in Latvia. In the light of this, we examine the harmonisation of practice and education in Latvia with EU norms.

## 1. Introduction

The PHARMINE (“*Pharmacy Education in Europe*”) consortium surveyed the state of pharmacy practice and education in the member states of the European Union (EU), including Latvia, between 2008 and 2011, with an update in 2017. The methodology used in the PHARMINE study and the principal results obtained have already been published [1]. 

In the first part of the study, PHARMINE gathered information on general, community practice, on specialised hospital and industrial practice, and on education and traineeship for pharmacists. PHARMINE also dealt with other personnel working in pharmacies such as pharmacists’ assistants: their education, training and responsibilities.

PHARMINE went on to study the legal and administrative context of pharmacy practice and education. In the EU—in contrast to other parts of the world—pharmacy practice and education fall under two jurisdictions: national and European. The latter is confederal both in structure and decision-making. Freedoms of movement, of residence, and of exercise of profession are the cornerstones of the EU. Thus, there is a system of automatic recognition of professional qualifications for seven sectoral professions (nurses, midwives, doctors, dentists, pharmacists, architects and veterinary surgeons). To work in another EU member state, professionals apply to the relevant authority of that country, providing proof of the qualifications (for harmonised EU practice) obtained in their home state. For sectoral professions, the European Commission of the EU issues directives; the latter are ordinances, laying down the broad imperatives, on the practice and education of the given profession [2]. An EU directive requires member states to achieve a particular result—in this case harmonisation of practice and education—without dictating the means of achieving that result. Directives leave the different member states with leeway as to the exact rules to be adopted. Member states may organise systems that are more or less harmonised with the EU paradigm. 

In parallel to the above pan-national system, member states may introduce specific national legislation relating to specialised practice, and to ownership and management of pharmacies, for example. 

This paper looks at how EU legislation impacts on pharmacy practice and education in Latvia.

Pharmacy education and training in Europe is also influenced by the Bologna agreement on the harmonization of European degree courses, and student and staff exchange [3]. The Bologna agreement, signed by the education ministers of the governments of the European Higher Education Area (48 members including the 28 EU member states), proposes recommendations that are not legally binding. They include a harmonised structure for all university degrees (including pharmacy) with a bachelor (3 years) followed by a master’s (2 years) degree. Here, the Bologna agreement is in opposition to the EU directive. The latter requires a five-year, “tunnel” degree structure for pharmacy, i.e., a degree course that offers no possibility for intermediate entry or exit after (successful) accomplishment of a three-year bachelor period. The idea behind the Bologna recommendation on degree structure is to improve student mobility. Mobility is also behind other Bologna recommendations such as the development of tools to promote student exchange programmes like the European Credit Transfer and Accumulation System (ECTS). This provides credits to students for defined learning outcomes and their associated workload. ECTS are coupled with the Diploma Supplement that describes the nature, level, context, content and status of the studies that were successfully completed by a student at a given university. This system allows students to validate studies carried out at their host university by their home university. 

This paper looks at how the Bologna process has developed in Latvian universities. It is particularly interesting to examine how this affects education and practice in a country, Latvia, that has recently joined the EU (2004). 

In order to place pharmacy practice within the general health situation in Latvia compared to the EU, it can be noted that life expectancy at birth (Table 1) in Latvia is lower than (males) or equal to (females) the EU average of 79.4 years. Healthy life expectancy (EU average 70.2 years) is much lower in males and slightly lower in females. Furthermore, expenditure on health is much lower than the EU average ($3611 per capita). 

## 2. Design

Information was obtained from two pharmacy departments in Latvia: the University of Latvia (UL, Ruta Muceniece and Una Riekstiņa) and Riga Stradins University (RSU, Baiba Maurina and Vija Enina), which replied to questions on:

- pharmacy;
  ○ practice (community, hospital and industrial);
  ○ legislation;
  ○ education and training;
- harmonisation with the EU sectoral directive on pharmacy;
- harmonisation with the Bologna recommendations (organisation of the degree course with the existence or not of a bachelor/master’s structure, implementation of ECTS and the Erasmus programme on student and staff exchange [6].

An electronic survey methodology was used and data was collected in 2010 and revised in 2017. We attempted at all times to collect objective data.

The information is presented in the form of tables in order to facilitate legibility. This type of presentation was developed in association with the journal’s editorial board and has been described in detail in a previous publication [7]. This format will ease the comparison of different EU countries by students and staff envisaging exchange programmes, and by researchers in pharmacy education and practice.

## 3. Evaluation and Assessment

### 3.1. Organisation of the Activities of Pharmacists, Professional Bodies

Table 2 provides details of the numbers and activities of community pharmacists and pharmacies in Latvia. Items are expounded in the “comments” column.

The data in Table 2 shows that compared to the EU linear regression estimation (for definition and calculation see Reference [1]), the ratio of the actual number of community pharmacists in Latvia (/population) compared to the linear regression estimation for Latvia = 1.09. Thus, the number of pharmacists per population is similar to the EU norm. The same comparison for community pharmacies produces a ratio of 1.24, above the EU norm. The use of the linear regression estimation is based on the following. Results here and elsewhere are not normal in distribution but highly skewed [1]. Skewness was due to the uneven distribution of population in the EU. A small proportion of the population of the EU lives in 18 smaller countries: Austria, Belgium, Bulgaria, Croatia, the Czech Republic, Denmark, Estonia, Finland, Greece, Hungary, Ireland, Latvia, Lithuania, Malta, Portugal, Slovakia, Slovenia and Sweden, and the larger proportion in 8 larger countries: France, Germany, Italy, the Netherlands, Poland, Romania, Spain and the United Kingdom. In countries with a larger population, the numbers of pharmacists, etc. are greater. As the number of countries with a small population is large this leads to skewness in the distribution. Therefore, we used an EU linear regression estimation to compare the data for a given country with the EU average. This was calculated taking the numbers of pharmacists, etc. as the dependent variable with the population as the independent variable. The reported number for the country was expressed as a ratio of the number estimated from this linear regression. As the comparison is now with transformed (corrected for population) data that are originally skewed, parametric tests such as the *t*-test with *p* values are not given.

The activities and occupations of pharmacists in Latvia are similar to those of community pharmacists in other EU member states [1]; a specificity of Latvia is the existence of internet pharmacy practice. 

Table 3 provides details of the numbers and activities of persons other than pharmacists working in pharmacies in Latvia.

Pharmacists’ assistants work under the supervision of the pharmacist. The legal definition of the profession states: “A pharmacist’s assistant is a health care provider, a specialist who works in pharmacies under the supervision of a pharmacist, and independently may sell health care or body care products, prepares drugs for individual doctor prescriptions or medical institution written requests (extemporal drugs). A pharmacist’s assistant works in wholesale markets and medicines manufacturing plants and carries out duties according to the specifics of the work” [12]. The training and functions of pharmacists’ assistants are harmonised with those in other EU member states [1].

Turning to specialisation in pharmacy practice, Table 4 provides details of the numbers and activities of hospital pharmacists in Latvia.

The number of pharmacists working in hospitals is similar to the EU average. The ratio of the actual number compared to the linear regression estimation is 1.23, (for definition and calculation see Reference [1]). The ratio for hospital pharmacies compared to the EU average is 0.82.

Table 5 provides details of the numbers and activities of industrial pharmacists and pharmacists in other sectors, in Latvia.

Industrial pharmacists in Latvia have similar practices and duties to those in other EU countries [1]. As accurate numbers of industrial pharmacists were not available for most European countries, a comparison with the EU average is not possible. The pharmaceutical market is loss-making in that imports are greater than exports. In 2015, the pharmaceutical trade balance was negative in the majority (16/28) of EU countries including Latvia [24]. The Latvian figure of −€178 million is far less than in some other member states such as Spain (−€2892 million) and Italy (−€2320 million). Other comparisons can be found in Reference [24]. This situation is relatively stable as figures for 2008 [24] show that 15/27 EU countries have a negative pharmaceutical trade balance with Latvia at −€160 million, Spain at −€1710 million and Italy at −€1714 million.

Table 6 provides information on professional associations for pharmacists in Latvia.

### 3.2. Pharmacy Faculties, Students, and Courses

Table 7 provides details of pharmacy higher-education institutions (HEIs), staff and students in Latvia.

Comparison to the EU average for ratios such as students/staff is difficult given the Latvian situation in which both staff and students can be either full-time or part-time and staff may be attached to the pharmacy department or to another university department. 

Table 8 below contains details of specialisation electives.

Table 9 provides details of past and present changes in pharmacy education and training in Latvia.

The main changes envisaged are the further alignement with EU norms and the introduction of outcomes-based learning of elements in English.

### 3.3. Teaching and Learning Methods

Table 10 provides details of hours by learning method (for further details on the definitions of the different methods see Reference [1]). 

The pharmacy programme of UL consists of obligatory and elective courses and includes lectures, seminars, presentation of reports, practicals, and presentation of research projects. Lectures, tutorials and practicals are given in the first 4 years (3 years bachelor programme and 1 year master’s programme with additional work on thesis). Electives are of 2 kinds: compulsory electives are pharmacy-oriented; free-choice electives may be from other programs and other faculties, and can be from the humanities. Traineeship is carried out in the first and second year of master’s programme years. The main components of the courses are lectures (24%), practicals (18%) and project work (24%), with traineeship at 19%. The bachelor thesis provides 15 ECTS and corresponds to 2.5 calendar months; it is an individual research project and so how many hours one needs to work depends on the chosen topic; an estimation is given in Table 10. In the same way, the master’s thesis cannot be calculated in hours. It provides 30 ECTS and corresponds to 5 calendar months or 1 semester. It also is an individual research project. Traineeship is at the end of the programme before the master’s thesis and can be carried out at community pharmacies or 3 months in a community pharmacy plus 3 months at a hospital pharmacy. It is also possible to do 3 months at a pharmacy in another country under the ERASMUS training programme [6] plus 3 months in a Latvian pharmacy.

At RSU there is a substantial number of practical hours (67.5%) that consist of practical work in a laboratory in contact with teaching staff and student’s individual work. Lectures and electives are validated at a session of the faculty committee and then by the University Senate, as is the traineeship that occurs in the 5th year and occupies 13% of the time.

### 3.4. Subject Areas

Table 11 provides details of student hours by subject area (for further details on the definitions of the subject areas see Reference [1]). Student hours are presence hours, not student workload hours. The generic area includes all issues that do not belong directly to a given appropriate course. For example, the competence to find and read scientific literature, to use library and internet data basis, to make presentations and develop presentation skills, to use computer programs, to write short scientific literature reviews, to use languages (foreign and Latin), and to use simple laboratory techniques, common in all laboratories. For the generic area, an estimation is given as these hours do not constitute a separate course but are embedded in other courses.

Taking the MEDISCI/CHEMSCI ratio (950/770 = 1.23 for UL and 1,030/859 = 1.19 for RSU) as an indicator of the nature of the content of the M. Pharm. degree courses [30], it appears that both are balanced; elsewhere in the EU the courses of some member states, e.g., Ireland (ratio = 2.6) are oriented towards medicinal sciences [30]. It should be noted that in a pharmacy department attached to a medical faculty (UL) and a department that is independent (RSU), ratios are very similar, viz 1.23 and 1.19, respectively. 

Full details of the programmes are available on the website of the lists of the accredited study programmes at Latvian universities [31]. The difference in the total number of hours between this table and Table 10 is explained by the fact that Table 11 concerns, essentially, contact hours whereas Table 10 concerns student hours.

At RSU in each study year students have the opportunity to choose elective courses. The hours spent for each area depends on the student’s choice; for example, a student can increase his/her PHARMTECH knowledge by choosing “Drug registration” or his/her BIOLSCI knowledge by choosing “Pharmaco-genetics”.

### 3.5. Impact of the Bologna Principles [3]

Table 12 provides details the various ways in which the Bologna declaration impacts on the pharmacy HEIs of Latvia.

Regarding the accreditation of courses, when opening a new programme universities have to receive a license from the government ensuring that the methodologies and procedures of the external evaluation of the quality of the study programme comply with the standards and guidelines developed by the European Association for Quality Assurance in Higher Education [24]. Only licensed programmes can be opened and, after their first year, can apply for accreditation. The accreditation of programmes is obligatory. The maximal accreditation term is 6 years. After that, re-accreditation or closure follows. Each year a self-assessment of programmes is written and student feedback is gathered after each semester and after each course. The internal quality assurance of the faculty and university system complies with the standards and guidelines for quality assurance in higher education developed by ENQA [34]. The internal quality assurance system is implemented at the level of faculty and university and is built on the ENQA guidelines, which determine the procedures for approval and periodic evaluation of the programme and the degree awarded, student assessment, academic staff quality, training tools and resources to help students, as well as information systems and public information. Quality assurance is built on internal and external audits, election of the academic staff, and competition for administration positions. The implementation, objectives, and learning outcomes of the programme, as well as the learning process, resources, partnerships, and management systems are reviewed on a regular basis. All study programmes are constantly accredited and re-accredited.

As the courses are based on EU norms, harmonisation does not appear to be a major problem, whereas economic and language difficulties are obstacles to exchange.

### 3.6. Impact of European Union (EU) Directive 2013/55/EC [2]

Table 13 provides details the various ways in which the EC directive impacts on pharmacy education and training in Latvia.

Latvia mainly conforms to the different aspects of the EU directive with, notably, a tunnel degree. 

## 4. Discussion and Conclusions

Community pharmacies in Latvia sell Rx (prescription) and OTC (over-the-counter) medicines, and provide consulting and diagnostic services. 

Pharmacists study five years at one of two universities—UL and RSU. At UL, the programme is based on a bachelor plus master’s 3 + 2 years system; graduates receive a health sciences bachelor degree in pharmacy followed by a health sciences master’s degree in pharmacy. At RSU, after a seamless 5-years’ programme, graduates receive a pharmacist’s degree.

The pharmacy curriculum is organized according to the EU directive 2013/55/EU and has the required courses in medical, biological and pharmaceutical subjects, as well as courses in physics, languages, and social science. There is a six months’ traineeship in pharmacy at the master’s level, following the end of theoretical courses. Both the UL and RSU degree courses are thus adapted to the Bologna principles of student exchange under ERASMUS and other systems. In both courses, “fundamental” sciences such as CHEMSCI, PHYSMATH and BIOLSCI figure mainly in the first 3 years (Table 11), whereas more advanced “pharmaceutical” subjects such as MEDISCI are taught throughout the course i.e., including and up to the end of the 4th year. The 5th year is mainly dedicated to traineeship (Table 10) as in other EU member states [1]. This chronological coincidence amongst member states in the teaching of subject areas and in traineeship, within the 5-year course, should facilitate student exchange. The latter appears to be more impacted by economic and language problems than by harmonisation.

After three years of practice, university graduates receive a pharmacist’s certificate. Pharmacists may own and manage community pharmacies or work at community and hospital pharmacies. The Pharmacy Law of Latvia states that new pharmacies may be opened in the form of a pharmacist’s practice, joint practice, or a private company. A pharmacy belonging to a municipality has to be headed by a certified pharmacist. A closed-type pharmacy may be opened by a hospital or a day-care facility. After three years of practice, university graduates receive a pharmacist’s certificate. To obtain this certificate they have to pass an examination and the certificate is valid for 5 years. During this period, pharmacists have to continue their education and collect 200 academic hours from participation in professional courses, workshops, seminars, etc. After that, pharmacists may apply to the Certification Commission for prolongation of their certificate. In practice, new pharmacies are not being opened and the majority of existing pharmacies now belong to pharmacy chains. Recent amendments to the Pharmacy Law state that a general pharmacy may be established in the form of a pharmacist’s practice, a joint practice (a civil law company) or a capital company. If the owner is not a pharmacist, he/she must to conclude a contract with a certified pharmacist providing pharmaceutical care. If the pharmacy takes the form of a capital company either a pharmacist must be a shareholder of not less than 50% of the capital, or certified pharmacists must comprise not less than half of the board members.

Pharmacists’ assistants study for 2.5 years at RMC and RCMC; they are employed at community or hospital pharmacies but are not allowed to manage a pharmacy.

Individual specialisation is possible during the bachelor and master’s degree theses by choosing a specific laboratory for a thesis in an appropriate topic, and also by choosing the appropriate elective courses. Specialisation is not obligatory, and students may choose more practical pharmacy or clinical courses. RSU offers specialisation programmes in master’s level clinical pharmacy and industrial pharmacy. Pharmacists are primarily employed in community pharmacies. 

Coming back to the observation in the introduction that the health status and context in Latvia is below the EU average (Table 1), the PHARMINE survey shows that such an observation does not result from a lack of education or practice, which are similar to EU norms. It may be that the answer to this dilemma would be a re-orientation and extension of pharmacy services in areas such as diagnostics, pharmaceutical care, especially concerning chronic illness in the elderly, vaccinations, public health campaigns, etc. Community and hospital pharmacists actively participate in public health activities. For example, together with medical students they measure blood pressure and glucose levels of volunteers in city parks and trains. Pharmacies also organize open days together with distributors of medical devices, such as demonstrations on how to measure bone density, to control obesity, to calculate body mass index, and to evaluate hepatic steatosis. While there is still some way to go until the people of Latvia reach the average health level of European countries, pharmacy practice and education seem well adapted to help in this process. Finally it should be noted that many variables contribute to health disparities within and between countries. Pharmacy may play a part but other influences are much stronger. For example, the cost of health care and access to it are major determinants of health status.

## Figures and Tables

**Table 1:** Health statistics for Latvia [4,5].

Total Population	1,970,000
Life expectancy at birth m/f (years)	70/79
Healthy life expectancy at birth m/f (years)	58/68
Total expenditure on health per capita	$940

**Table 2:** Numbers and activities of Latvian community pharmacists and pharmacies [8,9,10,11,12,13,14,15,16,17].

Item	Numbers	Comments
Pharmacists	2111	Data from October 2017. 1,970,000 inhabitants/2111 comm. pharmacists = 933 inhabitants/pharmacist
Pharmacies	810	Inhabitants/pharmacy: 2432 Pharmacists/pharmacy: 2.61
Competences and roles of community pharmacists		The competencies of community pharmacists are: supplying prescription medicines; managing medicines for some ailments; giving advice on medicines; screening services (blood cholesterol, glucose, pressure, etc.).
Is ownership of a community pharmacy limited to pharmacists?	No	Currently ownership is not limited to pharmacists. According to the Latvian Pharmacy Law [12] a pharmacy may be established in the form of a: pharmacist’s practice; joint practice (a civil law company); capitalised company. When delivering pharmaceutical care in a pharmacy owned by a local government or a person who is not a pharmacist, the respective person shall enter into a contract with a certified pharmacist. An in-patient medical treatment institution or a daytime hospital may open a closed type of pharmacy. A pharmacy in the form of a capital company may operate if at least one of the following conditions has been complied with: no less than 50% of the shares in the capital company are owned by a pharmacist; no less than one half of the members of the management board of the capital company are certified pharmacists.
Rules on geographical distribution of pharmacies?	Yes	1 pharmacy per 2000 inhabitants; at least 500 m between each pharmacy that has an extemporaneous dispensing and/or a 24-h duty pharmacy service. An affiliate cannot be less than 5 km from the main pharmacy [17].
Are drugs and health care products available to the general public by channels other than pharmacies?	Yes	It is possible to buy food supplements, hygiene products, and medical devices in specialised shops.
	No	All medicines, bandages, specific plasters (silicon etc.) are available only in pharmacies.
	Yes	There is one e-pharmacy in Latvia. http://www.webaptieka.lv/en/ (opened November 2017). It is possible for customers from other EU countries to order non-prescription medicines and other products from this e-pharmacy.

**Table 3:** Numbers and activities of other personnel working in pharmacies in Latvia.

Item	Numbers	Comments
Are persons other than pharmacists involved in community practice?	Yes	Pharmacists’ assistants with a college education.
Their numbers	1601 (2017)	This is a regulated profession with a diploma based on the European Council Directive 92/51/EEC [18] on a second general system for the recognition of professional education and training.
		Pharmacy students, medical students and nurses who have not completed their higher-education institution (HEI) course, can be employed as technicians, i.e., support staff at a pharmacy. Such technicians are not registered and their number is not known.
Organisations providing and validating education and training		Pharmacists’ assistants are trained at Riga 1st medical college (RMC) and (since 2016) at the Red Cross Medical College (RCMC) where the first 28 students matriculated in 2016. RCMC is an institution under the supervision of RSU.
		The programme lasts 2.5 years.
		All programs are recognized by the Latvian Accreditation Centre (Higher Education Quality Evaluation Centre (HEQEC)) and evaluated by national and foreign experts [19].
Subject areas		RMC:
		CHEMISCI 240 h
		PHYSMATH 0
		BIOLSCI 274 h
		PHARMTECH 440 h
		MEDISCI 840 h
		LAWSOC 200 h
		GENERIC 760 (including traineeship in a pharmacy)
		ELECTIVE 200 h
		(For abbreviations see Reference [1]).
		The course has theoretical courses (1268 h), and personal work (1686 h).
		RCMC:
		CHEMISCI 180 h
		PHYSMATH 72 h
		BIOLSCI 72 h
		PHARMTECH 172 h
		MEDISCI 654 h
		LAWSOC 116 h
		GENERIC 728 (including traineeship)
		ELECTIVE 64 h
		(For abbreviations see Reference [1]).
Competences and roles		Extemporaneous drug preparation. Dispensing of non-prescription medicines under the guidance of a pharmacist. Dispensing of hygienic and cosmetic products.

**Table 4:** Numbers and activities of hospital pharmacies and pharmacists.

Item	Numbers	Comments
Does such a function exist?	Yes	There are approximately 31 hospital pharmacies in Latvia.
		Pharmacists working in hospitals have the same status as those working in community pharmacies. In general, hospital pharmacists do not have a specialised education but are simply pharmacists who work at hospital pharmacies. Thus, their title is defined by their place of work. Clinical pharmacists are defined by their education and function. Only 3 clinical pharmacists are at present working in hospital pharmacies. They receive a special education and are graduates of the clinical pharmacy master‘s degree program at RSU [20].
Number of hospital pharmacists	83	Pharmacists working at hospital pharmacies and those working at community pharmacies are registered in one single register. There are approximately 2111 registered pharmacists, of whom 83 pharmacists (and 34 pharmacists’ assistants) work in hospital pharmacies.
		There is a specific section for hospital pharmacists within the Latvian Pharmacists’ Society, and approximately 117 persons (83 pharmacists and 34 pharmacists’ assistants) are members of this section. The Latvian Pharmacists’ Society is a member of the European Association of Hospital Pharmacists EAHP [21].
Competences and roles of hospital pharmacists		Purchasing of drugs and medical material. Unit-dose drug distribution. Production of patient-specific medicines (e.g., cytotoxic preparations).

**Table 5:** Numbers and activities of industrial pharmacists and pharmacists in other sectors.

Item	Numbers	Comments
**Industrial Pharmacy and Pharmacists**		
Pharmaceutical research and development (R&D) and production		Approximately 100 representative offices of foreign drug manufacturers were registered in Latvia, together with 29 local drug manufacturers and 84 drug wholesalers (2016) [22]. There are 84 drug wholesalers; the 5–6 larger companies have a licence to repack medicines or to label according to good manufacturing practice (GMP) [14]. The Latvian medicine manufacturers‘ market makes up 4.7% of the total medicine market [23]. The largest local generic producers are: Grindex: https://grindeks.eu/ Olainfarm: http://www.olainfarm.lv/eng/ LMP: http://www.lmp.lv/eng/products.php Pharmidea: http://www.pharmidea.lv/en/home/ Silvanols: http://www.silvanols.lv/ Olainfarm and Grindex are also licensed to produce and export original medicines. Pharmaceutical sales were €375 million (2016), in 2015 imports of €510 and exports of €332 million [24]. There is, thus, net importation of pharmaceuticals.
Number of pharmacists working in industry	200–300	1971 (2017) persons work in the Latvian pharmaceutical industry [24]. Approximately 200–300 pharmacists are working in the industry but there are no statistics in Latvia as registration is not required. 69 persons are members of the Industrial Pharmacists’ Section (IPS) section of the Pharmacists Society of Latvia (IPS) Industrial pharmacists are represented by the IPS [13]. IPS has been a member of the European Industrial Pharmacists’ Group (EIPG) since 2007 [25].
Competences and roles		Industrial pharmacists work together with health authorities in: drug laboratories; agencies (registration, expertise, etc.); production (qualified person (QP), etc.); representative offices; drug wholesalers (QP, responsible persons, etc.). Researchers with a scientific doctoral degree working in drug laboratories are not included in the register of pharmacists and are not called industrial pharmacists. A pharmacy degree is not obligatory for employment in R&D laboratories. Some pharmacy graduates may work in preclinical research, but again they are not registered and certified pharmacists. Since 2015 there has been specialisation in Industrial Pharmacy delivered as a joint programme between RSU and Riga Technical University. Generally, pharmacists in industry have one of the following posts: pre-clinical drug evaluation (safety and efficacy); (exceptionally) clinical drug evaluation; marketing; distribution; medical devices; cosmetology; drug evaluation and registration (governmental and industrial).
**Pharmacists Working in Other Sectors**		
Sectors in which pharmacists are employed		Pharmacists are employed by the national health authorities and in public sector agencies (e.g., the the drug pricing agency National Health Service (NHS) of Latvia, Health Inspectorate of Latvia, State Agency of Medicines of Latvia working on expertise of medicine dossiers and registration, and in veterinary and agriculture departments, forensics, etc.). Others are employed in the private sector in different areas (laboratories, representative offices for foreign drug firms, medical journalism, etc.). Some are employed by the HEIs.
Competences and roles in other sectors		Education and training, research, management, control, production, consulting, drug evaluation and registration.

**Table 6:** Professional associations for pharmacists in Latvia.

Item		Comments
Registration of pharmacists	Yes	The Pharmacists’ Society of Latvia (PhSL) is the only professional organisation in Latvia representing pharmacists and is responsible for the registration of pharmacists and pharmacists’ assistants [8]. The Latvian government also authorises the PhSL to perform the certification of pharmacists. This is obligatory for pharmacy managers but voluntary for other pharmacists.
Creation of pharmacies and control of territorial distribution	No	The creation of community pharmacies is under the responsibility of the licensing section of the State Agency of Medicines [14]; the territorial distribution of pharmacies follows the rules of the Cabinet of Ministers controlled by the Health Inspectorate.
Ethical and other aspects of professional conduct	Yes	There is an ethical code for pharmacists issued by the PhSL. The society has an ethical commission; their decision is irrevocable. Sanctions are: instruction, notification or annulment of the pharmacist certificate. For the pharmacy manager and owner, this implies penalties.
Quality assurance and validation of university courses	Yes	During the programme accreditation (and re-accreditation) by LATAK (Latvian National Accreditation Bureau) [26] an expert representative of the PhSL is invited. The PhSL has an educational section. They collaborate with both UL and RSU.

**Table 7:** Pharmacy higher education institutions (HEIs), staff, and students in Latvia [27].

Item	Number	Comments
Number of pharmacy HEIs in Latvia	2 + 2	Universities: University of Latvia (UL)
		RSU (RSU)
		Colleges for pharmacists’ assistants (see Table 3 and [26]):
		Riga Medical College No.1 (RMC)
		Red Cross Medical College (RCMC) of RSU (since 2016–2017)
Public pharmacy HEIs	2	There are no private pharmacy HEIs in Latvia.
Faculty attachment		UL: medicine
		RSU: independent.
Do HEIs offer bachelor and master degrees?	UL: yes RSU: no	In both the main HEIs there is a 5-year degree. UL: the master‘s degree programme in pharmacy is a continuation of the pharmacy bachelor programme (model 3 years + 2 years). RSU: three programmes are related to pharmacy: professional study programme in Pharmacy (5 years). Following the professional programme, students acquire a pharmacist’s degree equivalent to a master’s degree allowing them to work in a pharmacy; professional study programme for master’s degree in Health Care (2.5 years) (sub-division—clinical pharmacy); professional study programme in Industrial Pharmacy (1.5 years) leading to an Industrial Pharmacist’s professional qualification (RSU and Riga Technical University joint programme)**.**
**Teaching staff**		
Staff (UL)	UL: 12+19 RSU: 33	UL: the faculty of medicine teaching staff is composed of 130 persons (elected part-time or full-time teaching staff) and approximately 70 freelance teachers (Latvian nationals and guest lecturers from foreign countries), with 12 persons full- or part-time in pharmacy of which 6 are faculty of medicine professors. Nineteen other part-time members of the teaching staff in the pharmacy programme (8 professors and associate professors) come from various other faculties (chemistry, biology, physics, economics, modern languages, etc.). RSU: The faculty of pharmacy teaching staff is composed of 33 persons (Latvian nationals) (8 professors, 12 assistant professors, 13 lecturers and assistants). The teaching staff involved in the pharmacy study programme (70 in all) is composed also of staff from various other faculties (medicine, modern languages).
Professionals from outside the HEIs	UL: 4 RSU: 10+	UL: pharmacists from community pharmacies, scientific institutions, the Latvian State Health agency and from the hospital pharmacy. These 4 are lecturers invited to give academic courses. All certificated pharmacists from community or hospital pharmacies may act as tutors for pharmacy student trainees in pharmacy practice. Students have a free choice of the pharmacy for their traineeship. Usually tutors for trainees are from the main pharmacies in the capital city of Riga. Approximately 50–60 certified pharmacists have been mentors for students during the last 6 years. RSU: pharmacists from community pharmacies, hospital pharmacies, the pharmaceutical industry (especially the Industrial Pharmacy programme), professionals from the Laboratory of Pharmaceutical Pharmacology of the Latvian Institute of Organic Synthesis. There are approximately 10 invited lecturers for academic courses in academic year. Each student during study may have 3–5 pharmacists responsible for his/her traineeship.
**Students**		
Number of places on entry following secondary school	UL: 65 RSU: 48	UL: 65 full-time places—10 financed by the state budget and 5 financed by UL, 50 financed by private or legal persons. However, the number of matriculated students is less than the places offered. Gymnasium graduates apply simultaneously for several programmes at different universities and in the end choose a programme where state-budget places are available. RSU: (2016) 48 full-time places—40 financed by the state budget, 8 financed by private or legal persons, and 20 part-time places (following pharmacists’ assistant education) financed by private or legal persons.
Number of applicants for each entry place	UL: 353 RSU: 161	UL: (2017, statistics [28]) 248 applicants for budget places; 105 for self-financed studies. 16.53 applicants/state-budget financed place. For self-financed places there is no competition. RSU: 161 applicants for full-time; 17 for part-time studies. 4.03 applicants/place [29]
Specific pharmacy-related entrance examination.	No	UL: requires a General Certificate of Secondary Education with good results in the centralised national examination in chemistry (A–D level), Latvian and foreign languages. Assessment in chemistry is the determinant. RSU: requires a General Certificate of Secondary Education and centralised national examination in chemistry, Latvian and a foreign language. The main admission requirement is the assessment in the centralised examination in chemistry. In case of equal number of points, competition is determined by the centralised examination assessment in Latvian and in the foreign language. Part-time students are admitted on the basis of the average grade of their secondary school and 1st Medical College certificates.
Graduates that become registered pharmacists.		UL: masters programme graduates and graduates from the RSU pharmacy programme become registered pharmacists if they work in pharmacies. In other work places registration is not obligatory. About 90% of graduates start to work in pharmacies. Later some of them change their working place. RSU (2017): 26 full-time graduates and 1 part-time graduate, 2 industrial pharmacy graduates.
**Advanced entry**		
Entrance after a first bachelor year.	Yes	UL: the details of the study programme, study courses, credit points acquired the first HEI are evaluated. RSU compares the content and volume of the study courses with appropriate study courses in the RSU pharmacy programme and decides which courses can be accepted and which courses need additional examinations.
**Fees per year**		
For home and EU students	For UL and RSU: for full-time home students the fee is 2000 € per year, for UL the state budget place financing is €3386 for bachelor studies and €3558 for master‘s studies; for RSU state budget place financing for pharmacy studies is €4475; for part-time home students €1620 per year.	
For non-EU students	UL: bachelor programme: €4800/year, master‘s: €5660/year RSU: 1st and 2nd year—€8000 per year, 3rd, 4th and 5th year—€9000 per year	

**Table 8:** Specialisation electives in pharmacy HEIs.

Item		Comments
Do HEIs provide specialised courses?	Partial	The UL pharmacy programme (bachelor plus master‘s) and the RSU 5-year pharmacy programme are aligned with practice in community pharmacies. Thus the main “specialisation “ is pharmaceutical care. Pharmacists can obtain other individual skills in other life science branches or during the ERASMUS exchange programme. In addition to the above, RSU has 2 specialisations: clinical pharmacy; and industrial pharmacy.
Specialisation provided by other means?		RSU teaches courses in subjects such as hospital pharmacy, industrial technology of drug forms, etc., but these do not lead to a recognised specialisation diploma. RSU has opened two programmes for pharmacist specialisation: clinical pharmacy master‘s degree programme; and (recently) together with Riga Technical University professional Industrial pharmacy a 1.5-year programme.

**Table 9:** Past and present changes in education and training in Latvian pharmacy HEIs.

Item		Comments
Have there been any major changes since 1999?	Yes	UL: the pharmacy programme was opened in 2000 and therefore it was constructed according to the Bologna Declaration as two programmes—bachelor and master‘s degree programmes with an ECTS credit point system, a diploma supplement, traineeship of 6 months in the 2nd year of master‘s studies, compulsory and elective subjects, and a student’s research project. RSU: Introduction of outcomes-based pharmaceutical education. Revision of pharmacy study programme, implementation of such study courses as clinical trials, placement in industrial pharmacy, placement in hospital pharmacy, four National Degree exams instead of two, one in pharmaceutical care. Transformation of Clinical Pharmacy study programme in 2013 from academic (2 years) to professional course (2.5 years) by implementing six-month placement in clinical pharmacy in hospitals. New joint study programme in Industrial Pharmacy since 2015. 2016: the Academic Information Centre of Latvia started the implementation of the European Science Foundation project “Support for Meeting the Requirements Set for EQAR (European Quality Assurance Register for Higher Education) Agency”. The aim of the project is to ensure support for meeting the requirements set by EQAR, including raising the quality of the agency and strengthening its capacity. RSU participated in the project regarding the accreditation of the management of studies for Health Care. In 2017 the expert team recommended the accreditation of the study direction Health Care for all pharmacy programmes of RSU for a term of 6 years.
Are any major changes envisaged before 2019?	Yes	UL: Introduction of outcomes-based pharmaceutical education Improvement of the assessment system. Introduction of e-learning in full-time studies and part of courses in English. RSU: the implementation of the recommendations of the expert team: “It is recommended to recruit additional younger academic staff with good command of English. The study infrastructure should be further improved along with the material technical provisions at laboratories. The quality assurance system of the pharmacy programme with low student feedback response rates needs more transparency and revision. Ensure a consistent and fair approach to the allocation of credit points.” Mapping of study programmes, study course outcomes, with pharmacist’s professional standard.

**Table 10:** Student hours by learning method: University of Latvia (UL, top), Riga Stradins University (RSU, bottom).

**UL**							
**Method**	**Bachelor Programme Year 1**	**Year 2**	**Year 3**	**Master‘s Programme Year 1**	**Year 2**	**Total**	**%**
Lecture	300	400	250	300	0	1250	24
Tutorial	20	20	0	50	0	90	2
Practical	200	200	250	250	0	900	18
Project (bachelor thesis 400 h and master‘s thesis 800 h)	0	0	400	0	800	1200	24
Traineeship (6 months)	0	0	0	160	800	960	19
Electives	200	100	60	120	0	480	9
Optional	80	80	40	0	0	200	4
**Total**	800	800	1000	880	1600	5080	100
**RSU.**							
**Method**	**Year 1**	**Year 2**	**Year 3**	**Year 4**	**Year 5**	**Total**	**%**
Lecture	190	130	187	126	0	633	9
Tutorial	47	32	44	46	0	169	2
Practical	540 + 540	561 + 561	598 + 598	724 + 724	160	5006	67.5
Project	0	0	0	0	320	320	4
Traineeship (hospital)	0	0	0	0	54	54	0.7
Traineeship-community	20	0	0	0	648	668	9
Optional/electives	80	160	80	160	80	560	8
**Total**	1417	1444	1507	1780	1262	7410	100

**Table 11:** Student hours by subject area: University of Latvia (UL, top), Riga Stradins University (RSU, bottom).

**UL**							
**Subject Area**	**Bachelor Programme Year 1**	**Year 2**	**Year 3**	**Master‘s Programme Year 1**	**Year 2**	**Total**	**%**
CHEMSCI	250	250	200	70	0	770	15
PHYSMATH	0	50	0	50	0	100	2
BIOLSCI	100	100	0	100	0	300	6
PHARMTECH	50	100	200	100	0	450	9
MEDISCI	250	300	200	200	0	950	19
LAWSOC	50	0	0	100	0	150	3
GENERIC	100	0	0	100	0	200	4
TRAINEESHIP	0	0	0	160	800	960	19
PROJECT	0	0	400	0	800	1200	24
Total	800	800	1000	880	1600	5080	100
**RSU**							
**Subject Area**	**Year 1**	**Year 2**	**Year 3**	**Year 4**	**Year 5**	**Total**	**%**
CHEMSCI	270	273	160	156	0	859	22
PHYSMATH	72	0	40	0	0	112	3
BIOLSCI	160	60	40	0	0	260	6
PHARMTECH	24	0	392	176	6	598	15
MEDISCI	164	382	184	250	50	1030	26
LAWSOC	32	40	0	144	0	216	6
GENERIC	40	0	0	60	0	100	3
TRAINEESHIP	20	0	0	20	702	742	19
Total	782	755	816	806	758	3917	100

CHEMSOC: chemical sciences; PHYSMATH: physical and mathematical sciences; BIOLSCI: biological sciences; PHARMTECH: pharmaceutical technology; MEDISCI: medicinal sciences; LAWSOC: law and social sciences; GENERIC: generic competences.

**Table 12:** Ways in which the Bologna declaration impacts on Latvian pharmacy HEIs.

Item		Comments
“Comparable degrees with diploma supplement”	Yes	UL: since 2004. Students receive a diploma supplement. RSU: together with their pharmacy diploma graduates also receive a diploma supplement in Latvian and English.
“Two main cycles (B and M) with entry and exit at B level”	UL: Partial RSU: No	UL has a 3-year bachelor degree which leads automatically to a 2-year master‘s degree.
“European Credit Transfer System (ECTS) system of credits with links to life-long learning (LLL)”	Yes	UL: national credit points (CP) are linked to ECTS and 1 CP corresponds to 1.5 ECTS in Latvian universities. ECTS are also given for all courses and shown in the course catalogue and in the diploma supplement. ERASMUS exchange students receive ECTS and UL validates ECTS obtained in other countries. RSU: the system is similar to that above. ECTS can also be acquired in a non-HEI context, e.g., students undergoing a traineeship in another member state will be assessed in ECTS.
“Addressing obstacles to mobility”		UL: mobility is organized within the framework of the ERASMUS exchange or training programme or with different fellowships. Non-ERASMUS mobility is possible through fellowships such as the Fulbright fellowship, fellowships from other universities, support from sponsors, etc. The university international office and persons in the faculty responsible for the ERASMUS exchange programmes help with housing and information; language courses are available. Every year 7–8 pharmacy students receive ERASMUS fellowships. The number of incoming students in the pharmacy programme from other countries is irregular. For UL students, the main mobility problem is finance and the fact that our students work in parallel to studying. For incoming students, the main problem is the Latvian language. RSU has a similar system. Language courses for students are also organised. Exchange is organised with the Netherlands, Germany, Portugal, Spain and Poland. Approximately 2–3 pharmacy students have the opportunity to receive an ERASMUS+ fellowship every year.
European/international quality assurance of courses	Yes	UL: the programme is evaluated by international experts and this involves site visits. The accreditation body is the Higher Education Quality Agency (AIKA) [32]. AIKA invites international and national experts to site visits and after that experts write their evaluation report. The final decision lies with the Council of Higher Education and the Accreditation Commission of the university. The study programmes were also evaluated by the European University Association [33]. RSU: a similar system with international experts is used (see Table 9).
European dimension	Yes	UL: the faculty of medicine has experience of teaching foreign students in the General Medicine programme, where 20% of the programme is in English; some of these students change and are enrolled in the pharmacy programme. Students attend summer schools when financially possible. Visiting researchers from Germany, USA and The Netherlands, etc. are invited to give lectures. Recently, courses (6 ECTS) in the bachelor programme and 12 ECTS in the master‘s programme have been given in English. RSU: our vision is a modern, prestigious university acknowledged in Europe and the world in the fields of healthcare and social sciences, with the human being at its centre of attention. RSU has international students from 53 countries (42% from Germany, 18% from Sweden.)
ERASMUS staff exchange to Latvia from elsewhere	Yes	UL: 1 staff month. Each year there is an exchange with Germany, Netherlands, Bulgaria, Italy. RSU: only short-term exchange (1 week) is possible.
ERASMUS staff exchange from Latvia to other HEIs	Yes	UL: 2 staff months. Each year there is an exchange with Italy, Hungary, Bulgaria and France. RSU: several persons (staff training and teaching visits) in the academic year 2016/2017.
ERASMUS student exchange to Latvia from elsewhere	Yes	UL: 8 student months. As degree studies are in Latvian, foreign students mainly do research and come on ERASMUS fellowships (from Ireland, Germany, The Netherlands, Sweden). RSU: 90 incoming students in 2017. In the pharmacy degree all courses are at the moment in Latvian, which can present a problem. RSU accepts students to do research projects within the framework of the ERASMUS programme, usually 2 incoming students in pharmacy per semester.
ERASMUS student exchange from Latvia to other HEIS	Yes	UL: Each year students go to Finland, Italy, Portugal, The Netherlands, Bulgaria, Estonia or Sweden. Courses that do not correspond to the UL programme are accepted as elective courses or research projects and validated according to credit points obtained abroad. RSU: 3 outgoing students in pharmacy in 2017. There is an ERASMUS exchange in pharmacy with the Netherlands, Germany and Italy.

**Table 13:** Ways in which elements of the European Commission (EC) directive (left column) impact on Latvian pharmacy HEIs.

Item	Comments
“Evidence of formal qualifications as a pharmacist shall attest to training of at least five years’ duration…”	This applies in Latvia. UL: globally we find this acceptable. In Latvia only pharmacists with a master‘s degree are registered as pharmacists. Persons with a bachelor degree in pharmacy can work in industry, government agencies, in research institutions, etc. However, 99% of pharmacy bachelors continue their studies in a master‘s degree programme. RSU: five years’ education is necessary for pharmacists to develop the required professionalism. Like other health care specialists, a pharmacist must work alongside medical specialists. At the same time, a pharmacist must be able to perform research in chemical and biological areas. Although a long 5-year duration makes a pharmacist’s education expensive, quality, professionalism, and the ability to keep up with the latest developments are the most important attributes.
“…four years of full-time theoretical and practical training at a university or at a higher institute of a level recognised as equivalent, or under the supervision of a university;”	This applies in Latvia. UL: a 4-year programme is acceptable given that with a study year of 10 months‘ duration and 6 months‘ training, plus a 2.5-month bachelor thesis and 5-month master‘s thesis, this leaves 26.5 months for theoretical and laboratory training courses. RSU: pharmacists must have the knowledge, skills and competences in many areas. It may be possible to provide a theoretical basis in four years, but not in a shorter time.
“…six-month traineeship in a pharmacy which is open to the public or in a hospital, under the supervision of that hospital’s pharmaceutical department.”	This applies in Latvia.
